# Comparative transcriptome profiling of a thermal resistant vs. sensitive silkworm strain in response to high temperature under stressful humidity condition

**DOI:** 10.1371/journal.pone.0177641

**Published:** 2017-05-18

**Authors:** Wenfu Xiao, Peng Chen, Jinshu Xiao, La Wang, Taihang Liu, Yunfei Wu, Feifan Dong, Yaming Jiang, Minhui Pan, Youhong Zhang, Cheng Lu

**Affiliations:** 1 State Key Laboratory of Silkworm Genome Biology, Key Laboratory of Sericultural Biology and Genetic Breeding, Ministry of Agriculture, Southwest University, Chongqing, China; 2 Sericultural Research Institute Sichuan Academy of Agricultural Sciences, Sichuan Nanchong, China; USDA Agricultural Research Service, UNITED STATES

## Abstract

Thermotolerance is important particularly for poikilotherms such as insects. Understanding the mechanisms by which insects respond to high temperatures can provide insights into their adaptation to the environment. Therefore, in this study, we performed a transcriptome analysis of two silkworm strains with significantly different resistance to heat as well as humidity; the thermo-resistant strain *7532* and the thermos-sensitive strain *Knobbed*. We identified in total 4,944 differentially expressed genes (DEGs) using RNA-Seq. Among these, 4,390 were annotated and 554 were novel. Gene Ontology (GO) analysis of 747 DEGs identified between RT_48h (Resistant strain with high-temperature Treatment for 48 hours) and ST_48h (Sensitive strain with high-temperature Treatment for 48 hours) showed significant enrichment of 12 GO terms including metabolic process, extracellular region and serine-type peptidase activity. Moreover, we discovered 12 DEGs that may contribute to the heat-humidity stress response in the silkworm. Our data clearly showed that 48h post-exposure may be a critical time point for silkworm to respond to high temperature and humidity. These results provide insights into the genes and biological processes involved in high temperature and humidity tolerance in the silkworm, and advance our understanding of thermal tolerance in insects.

## Introduction

Insects are traditionally considered as poikilotherms and the surrounding climatic components, such as temperature and humidity, strongly affect their behavior, metabolic rate, growth and development [1–3]. Therefore, temperature and humidity tolerance in insects are considered as important physiological traits that affect their adaptation to the environment. In order to overcome and/or minimize the deleterious effects of extreme high temperatures, insects have evolved a series of strategies, such as thermal tolerance [4], rapid heat hardening [5], protective dehydration [6] and diapause [7]. For the control of insect pests and the production of economic insects, it is important to clarify the thermal resistant mechanism of insects. Although previous studies have shown that heat shock proteins (HSPs) and antioxidants are involved in insect thermal resistance [8, 9], the potential mechanisms underlying thermotolerance of insects are less clear.

The domestic silkworm, *Bombyx mori*, is not only an important economic insect for silk production, but it is also an important model of lepidopterans [10], which are major pests of plants in agriculture and forestry sectors. With the completion of the silkworm genome [11], transcriptome [12], and the development of various platform technologies, the identification of key genes involved in important physiological processes has become more effective. During the breeding process, silkworms are affected by multiple environmental conditions especially temperature and humidity, which can directly affect the yield and quality of silk. Currently, China's sericulture is shifting from west to east, with the west having more hot and humid weather. To this end, the cultivation of high temperature and humidity-resistant silkworm strains is very urgent, and the identification of high temperature and humidity-resistant genes is particularly important. Although some studies in recent years have reported the tolerance-related genes in silkworm only a few functional genes have been identified to date [13–15].

**Fig 1:**
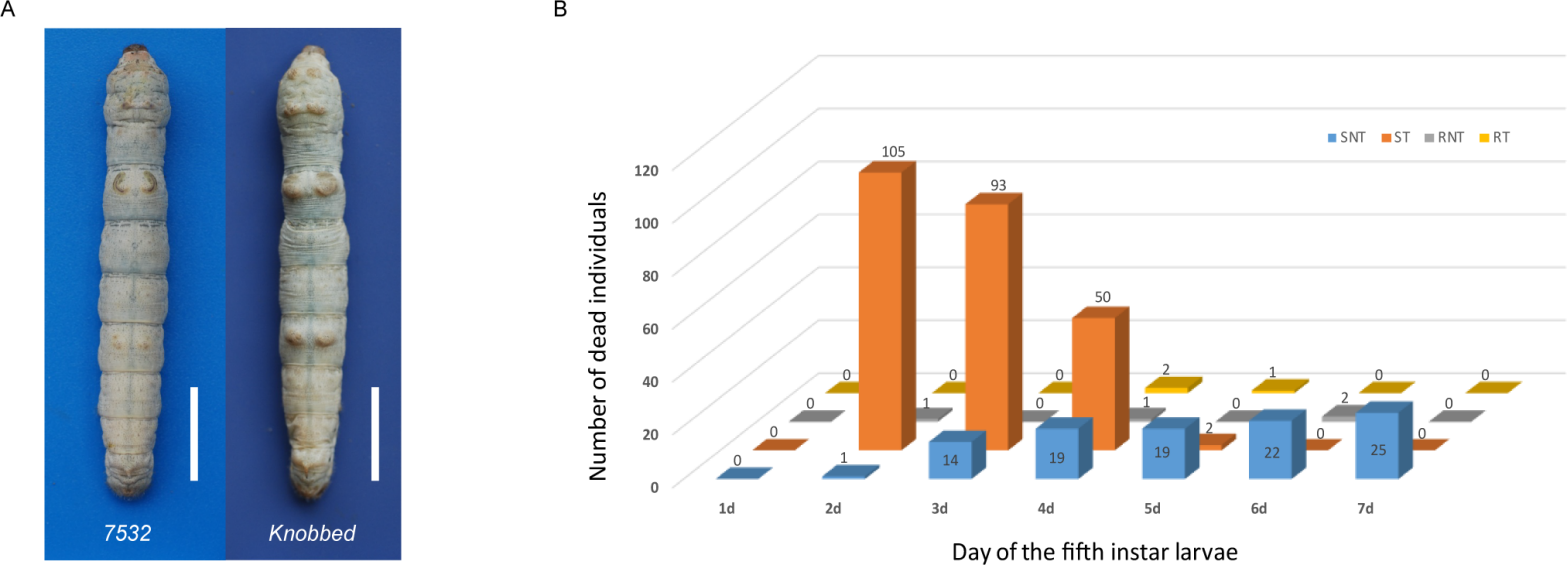
**Phenotype of fifth instar larvae (A) and numbers of dead *7532* and *Knobbed* larvae after high temperature and humidity treatments (B).** (A) Fifth instar larva from the thermo-resistant strain *7532* (left) and the susceptible strain *Knobbed* (right). Scale bars = 1 cm. (B) Larval deaths of the two strains were investigated every day from day 1 to day 7 in the 5^th^ instar. Numbers above each column represent the number of dead individuals. SNT, *Knobbed* strain without high temperature and humidity treatment; ST, *Knobbed* strain with high temperature and humidity treatment; RNT, *7532* strain without high temperature and humidity treatment; RT, *7532* strain with high temperature and humidity treatment.

Silkworm strains with resistance to different environmental conditions have provided the opportunity to identify the genes that regulate the corresponding resistant traits. In the present study, we used the silkworm thermo-resistant silkworm strain *7532* and the sensitive strain *Knobbed* for comparative analysis (Fig 1A). The *7532* is a polyvoltine strain, while the *Knobbed* is a bivoltine strain. To better understand the complex mechanisms underlying the resistance of silkworm strains to high temperature and humidity, we used RNA-Seq to generate comprehensive transcriptome profiles of *7532* and *Knobbed* after exposure to high temperature for different times under stressful humidity conditions. We then analyzed the differentially expressed genes (DEGs) among the different samples by using bioinformatics approaches and identified important genes as well as biological pathways related to thermotolerance in the silkworm.

## Materials and methods

### Silkworm strains

Two silkworm strains were used in this study: the thermo-sensitive strain, *Knobbed* and the thermo-resistant strain, *7532*. Developing embryos were incubated at 25°C with adequate humidity, and the first to fourth instar larvae were mixed reared with fresh mulberry leaves at 25°C and 70% relative humidity under a 12 h light/12 h dark photoperiod. We state clearly that no specific permissions were required for these locations/activities, because both strains were maintained by ourselves at the Sericultural Research Institute Sichuan Academy of Agricultural Sciences, China. We confirm that the field studies did not involve endangered or protected species.

### Treatments and samplings

On the first day of the fifth instar, the two strains *Knobbed* and *7532* were separately exposed to heat shock at 35 ± 0.5°C and 90 ± 2% relative humidity, while the control groups were maintained at 25 ± 0.5°C and 65 ± 2% relative humidity to increase the difference with the experimental groups. To ensure consistency in the environment, all treatments were conducted in a room with a temperature and humidity control system. The experimental as well as the control groups had 5 replicates each containing 50 randomly selected larvae derived from the same parent. In all groups, the number of larval deaths was observed at 9 AM each day. Surviving larvae in each experimental and control groups were collected after 1, 6, 24 and 48 h and were stored in liquid nitrogen until extraction of mRNA. The collected samples were named as follows: *Knobbed* with treatment (ST, Sensitive with Treatment), *Knobbed* without treatment (SNT, Sensitive Non-Treatment), *7532* with treatment (RT, Resistant with Treatment), *7532* without treatment (RNT, Resistant Non-Treatment).

### RNA preparation

Total RNA from the samples was isolated using TRIzol Reagent (Invitrogen, Carlsbad, CA, USA) according to the manufacturer’s protocol. Each sample consisted of five silkworm individuals. RNA degradation and contamination were detected using 1% agarose gels and RNA purity was assessed using a NanoPhotometer^®^ spectrophotometer (IMPLEN, CA, USA). RNA concentration and integrity were measured using Qubit^®^ RNA Assay Kit in the Qubit^®^ 2.0 Flurometer (Life Technologies, CA, USA) and the RNA Nano 6000 Assay Kit of the Bioanalyzer 2100 system (Agilent Technologies, CA, USA), respectively. A minimum RNA integrity number (RIN) of 6.8 was acceptable for a sample.

### Library construction and Illumina sequencing

Both cDNA library construction and Illumina RNA-Seq were carried out by Novogene (Beijing, China). A total of 3 μg RNA per sample was used as input material for the RNA sample preparations. Sequencing libraries were generated using the NEBNext^®^ Ultra™ RNA Library Prep Kit for Illumina^®^ (NEB, USA) according to the manufacturer’s protocol, and index codes were added to attribute sequences to each sample. The clustering of the index-coded samples was performed by a cBot Cluster Generation System with TruSeq PE Cluster Kit v3-cBot-HS (Illumina) according to the manufacturer’s instructions. After cluster generation, the library preparations were sequenced on an Illumina Hiseq platform, and 125 bp/150 bp paired-end reads were generated.

### Transcriptome data analysis

For quality control, raw data in FASTQ format were processed by in-house Perl scripts to obtain clean data [16]. Here, the reads containing adapter, reads containing poly-N and low quality reads for which the base number of Q_phred_ ≤ 20 is more than 50% of the entire read length were removed from the raw data. Meanwhile, the Q20, Q30 and GC content of the clean data were calculated. All the downstream analyses were performed with the clean data with high quality. The clean data were aligned to the reference genome downloaded from ftp://ftp.ensemblgenomes.org/pub/release-23/metazoa/fasta/bombyx_mori/dna/ with Bowtie v2.2.3 [17] and TopHat v2.0.12 [18]. For both software, the mismatch score was = 2 while maintaining default values for the remaining parameters.

The reads numbers mapped to each gene were counted using HTSeq (v0.6.1) [19] and the expected number of Fragments Per Kilobase of transcript sequence per Millions base pairs sequenced (FPKM) was used to estimate gene expression levels [20]. DEGSeq R package (1.20.0) [21] was used for the differential expression analysis, and the P values were adjusted using the Benjamini & Hochberg method. The threshold for significantly differential expression was used a set of corrected P value of 0.005 and log2(fold change) of 1. Venn diagrams were created using an online tool, Venny 2.1.0 (http://bioinfogp.cnb.csic.es/tools/venny/index.html) [22]. Gene Ontology (GO) enrichment was performed by the GOseq R package [23]. Kyoto Encyclopedia of Genes and Genomes (KEGG) (http://www.genome.jp/kegg/) is a database resource for systematic analysis of gene functions [24], and we used KOBAS software to test the statistical enrichment of differential expression genes in KEGG pathways [25].

### Validation of differentially expressed genes by quantitative real-time PCR (qRT-PCR)

The expression levels of DEGs were verified by qRT-PCR. TRIzol Reagent (Invitrogen) was used to isolate total RNA from each sample. Then, cDNA was synthesized from 2 μg total RNA from each sample using oligo(dT) primers and a Moloney murine leukemia virus reverse transcriptase (Promega, Madison, WI, USA) according to the manufacturer’s instructions. qRT-PCR experiments were performed using a CFX96 Touch Real-Time PCR System (Bio-Rad, USA) with iTaq™ Universal SYBR^®^ Green Supermix (Bio-Rad, USA). The total volume of qRT-PCR reactions was 10 μl each containing 5 μl iTaq™ Universal SYBR^®^ Green Supermix, 3 μl H_2_O, 1 μl cDNA template and 1 μl of gene specific primers. Sequences of primers used in qRT-PCR are listed in S1 Table. The *B*. *mori* eukaryotic translation initiation factor 4A (silkworm microarray probe ID: sw22934) was used as a reference gene. The qRT-PCR reaction conditions were as follows: 95°C for 30 s, followed by 40 cycles at 95°C for 5 s and 60°C for 34 s. Melt curves were obtained by increasing the temperature from 65°C to 95°C with increments of 0.5°C per 5 s. Relative expression was calculated using the 2^-ΔΔCt^ method. For each treatment, three biological replicates were used.

## Results

### Heat-humidity tolerance assays in *Knobbed* and *7532* larvae

During the preservation and investigation of silkworm resources, we found that the *Knobbed* strain is sensitive to high temperature and humidity, while the *7532* strain is resistant to high temperature and humidity. To evaluate and compare the heat-humidity tolerance of these two strains, larval deaths were investigated every day from day 1 to day 7 in the fifth instar (Fig 1B). For the sensitive strain *Knobbed*, 105 out of 250 individuals (42%) in the treatment group died on the second day, and almost all larvae died on the fourth day. In contrast, only 3 individuals (3 of 250) in total died in the resistant strain *7532* with all deaths occurring after the heat-humidity treatment. These results revealed that there is a significant difference between the two strains in their resistance to high temperature and humidity. In addition, our data also showed that the *7532* strain treated at high temperature and humidity moulted one day earlier than the control group (S2 Table), suggesting that the high temperature and humidity environment may can promote the development and metamorphosis of the *7532* strain.

### RNA sequencing and assembly

To screen genes related to heat-humidity tolerance through deep sequencing, we isolated mRNA from *Knobbed* and *7532* larvae collected at different time points (1, 6, 24 and 48 h) in the high heat-humidity treatment group and the controls. Thus, we constructed 16 transcriptome libraries and obtained 42.4 to 55.3 million raw reads for each library (Table 1). The sequenced raw data have been submitted to the NCBI Short Reads Archive (SRA) with the accession number SRP096246. The Phred Quality Score Q30 of each sample was at least 87.04%. A summary of transcriptome sequencing data was listed in Table 1 and the results demonstrated that the overall quality of our sequencing data was robust. After performing quality control, 6.00 to 7.82 Gb clean bases were obtained for each sample (Table 1), and 61.34%-74.32% clean reads were matched to the silkworm reference genome (S3 Table). In addition, a total of 2409 novel genes were identified based on the gene model annotation file downloaded from ftp://ftp.ensemblgenomes.org/pub/release-23/metazoa/gtf/bombyx_mori/ (S4 Table).

**Table 1:** Summary of transcriptome sequencing data.

Sample name	Raw reads	Clean reads	Clean bases	Error rate(%)	Q20(%)	Q30(%)	GC content(%)
RT_1h	46537730	44563956	6.68G	0.01	97.09	93.01	47.07
RNT_1h	47460878	45471478	6.82G	0.01	97.11	93.05	46.61
RT_6h	54393136	52161902	7.82G	0.01	97.12	93.1	46.69
RNT_6h	52715716	50700782	7.61G	0.01	97.33	93.45	44.78
RT_24h	55324830	50542622	7.58G	0.02	95.17	88.71	47.50
RNT_24h	54121840	49438268	7.42G	0.02	95.31	89.01	47.39
RT_48h	45608344	42586580	6.39G	0.02	96.14	91.27	48.69
RNT_48h	42402558	39971114	6.00G	0.02	96.52	91.88	47.42
ST_1h	47606712	45798296	6.87G	0.01	97.47	93.69	44.99
SNT_1h	47229400	45394444	6.81G	0.01	97.13	92.96	44.92
ST_6h	55283850	50579314	7.59G	0.02	95.72	89.64	45.44
SNT_6h	48306864	44136510	6.62G	0.02	96.07	90.47	44.24
ST_24h	51054876	46817034	7.02G	0.02	95.61	89.41	46.48
SNT_24h	53105260	48923266	7.34G	0.02	95.85	90.02	45.41
ST_48h	46346558	42895494	6.43G	0.03	94.45	87.04	46.84
SNT_48h	49788788	45688250	6.85G	0.02	95.51	89.33	46.41

**Note:** Q20 represents the percentage of bases with a Phred value >20. Q30 represents the percentage of bases with a Phred value >30. G represents gigabase.

### Differential gene expression profiles in *Knobbed* and *7532* after high temperature treatment

**Fig 2:**
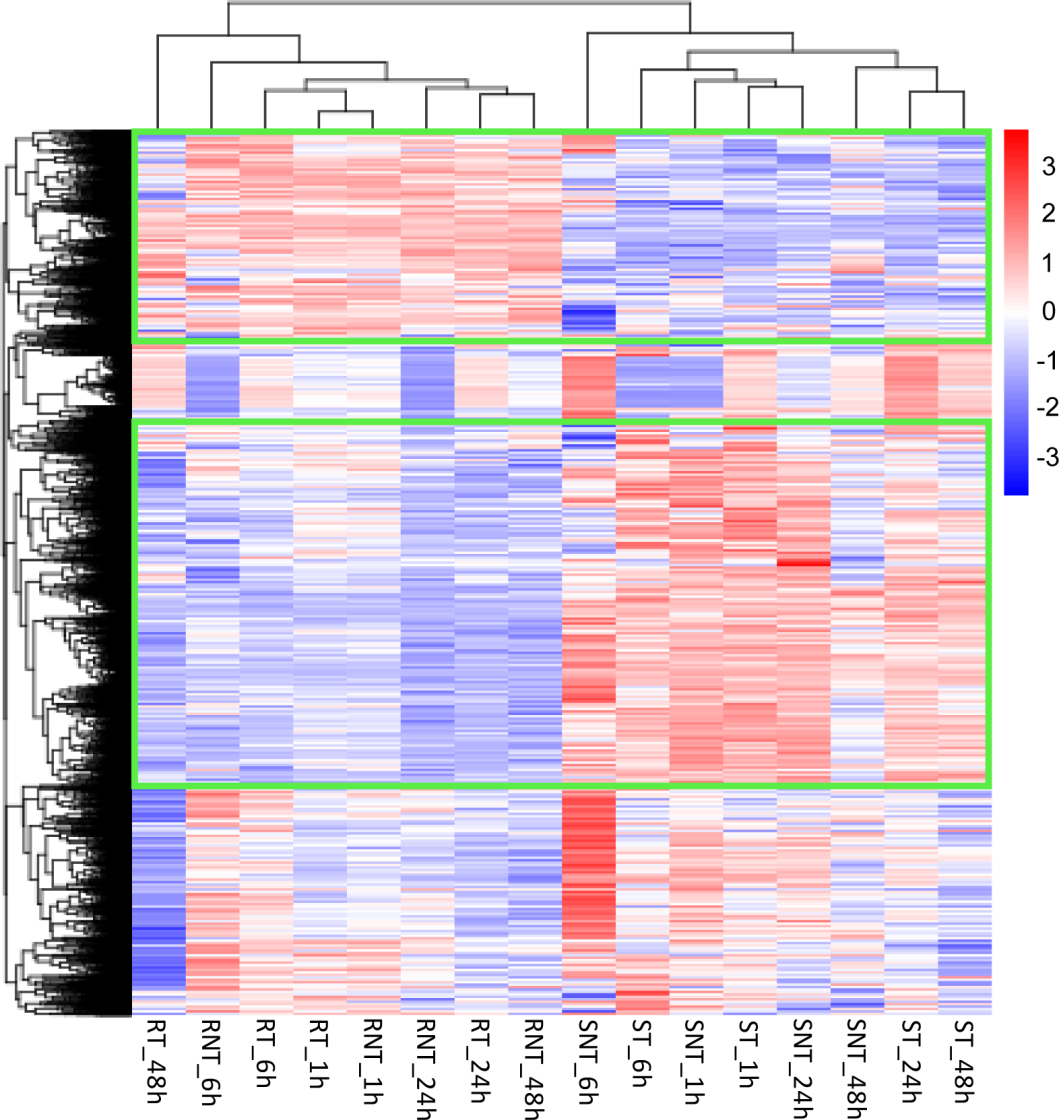
Hierarchical clustering graph of DEGs. Cluster analysis of the 4944 DEGs identified among the 16 samples by comparing any two samples based on log_10_(FPKM+1) values. Red bands represent high levels of gene expression; blue bands represent low levels of gene expression. The green boxes indicate DEGs regularly distributed between the *7532* and *Knobbed* strains. SNT, *Knobbed* strain without high temperature and humidity treatment; ST, *Knobbed* strain with high temperature and humidity treatment; RNT, *7532* strain without high temperature and humidity treatment; RT, *7532* strain with high temperature and humidity treatment; the numbers after these sample names represent the time after treatment; h, hour.

The transcription level for each gene was measured as the expected number of Fragments Per Kilobase of transcript sequence per Millions base pairs sequenced (FPKM) [20], and genes with FPKMs of more than 1 were considered to be expressed. Our data showed that more than 50% of the genes were expressed (FPKM ≥ 1) in each sample except RNT_24h (49.34%) and that more than 6.5% were highly expressed (FPKM > 60) (S5 Table). By comparing any two samples among the 16 samples, we identified a total of 4944 DEGs, including 4390 annotated genes and 554 novel genes (S6 Table). To understand the DEGs expression patterns, a hierarchical clustering analysis was performed with all samples based on log_10_(FPKM+1) values (Fig 2). The hierarchical clustered graph showed that different samples of the two stains clustered together. Moreover, a number of DEGs were regularly distributed between the different strains (green box) (Fig 2). Our results also showed that the differences in gene expression were the most obvious in the resistant strain 48 h after treatment, while for the sensitive strain it was evident at 6 h. The details of DEGs presented in the hierarchical clustered graph were shown in the S1 Fig. In addition, a clustered graph based on the treatment time between treated and control groups of the same strain was shown in S2 Fig and the details of DEGs were presented in S3 Fig.

### GO and KEGG pathway enrichment analysis of DEGs

**Fig 3:**
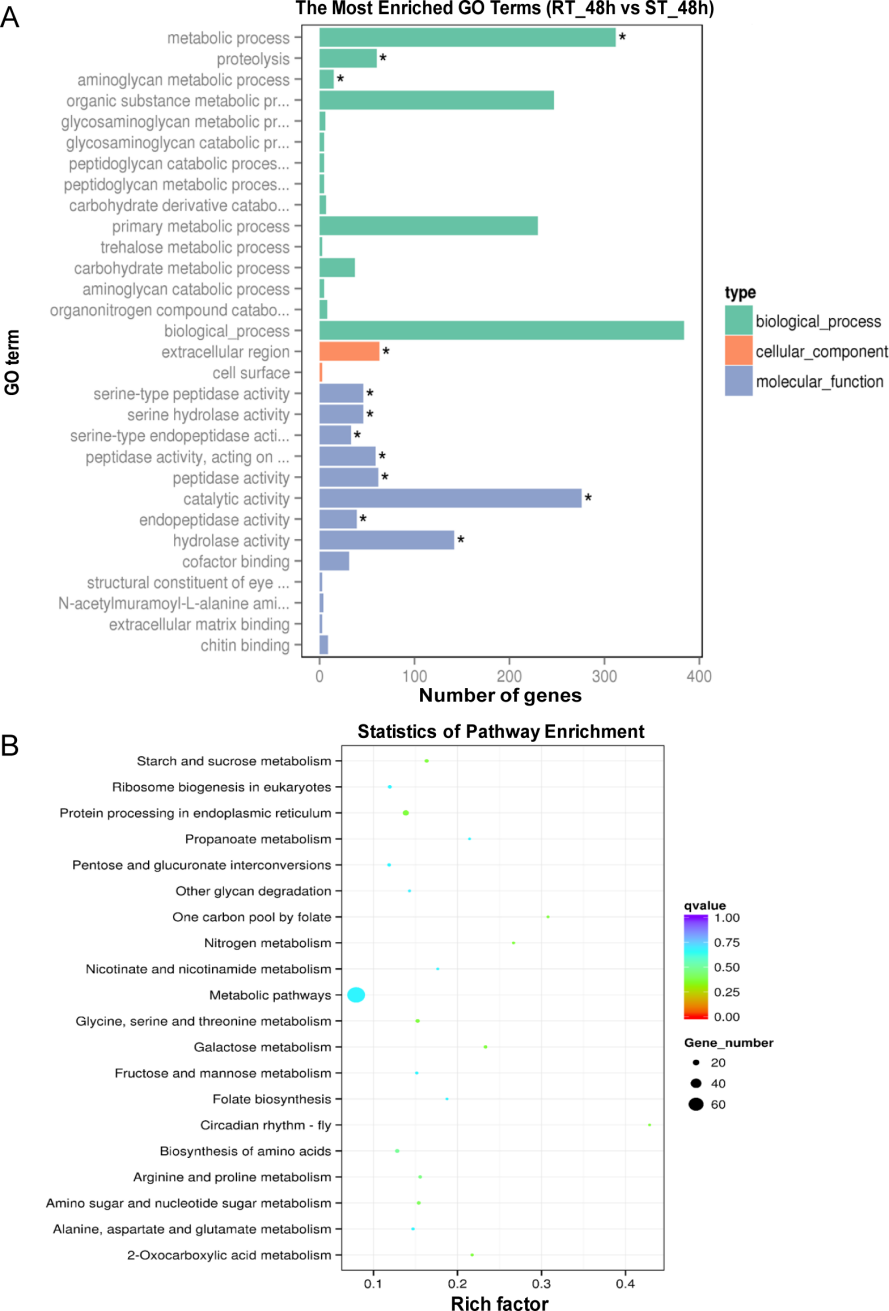
GO and KEGG enrichment analyses of DEGs. (A) GO enrichment analysis of DEGs obtained from comparing RT_48h and ST_48h. Asterisks indicate significantly enriched GO terms (Corrected_p Value < 0.05). (B) Scatterplot of enriched KEGG pathways for DEGs obtained from comparing RT_48h and ST_48h. The color and size of the dots represent the range of q values (Corrected_p Value) and gene number, respectively.

To better analyze the heat-humidity tolerance related genes, we performed GO and KEGG pathway analyses based on DEGs obtained from comparing the two strains after treatment for 48 h (RT_48h vs ST_48h), when the silkworms began to die under the high temperature. A total of 747 DEGs were identified between RT_48h and ST_48h (S7 Table), and were categorized into the three functional GO groups, biological process, cellular component and molecular function. The results showed that 12 GO terms were significantly enriched (Corrected_p Value < 0.05), including metabolic process, proteolysis, aminoglycan metabolic process, extracellular region, serine-type peptidase activity, serine hydrolase activity, serine-type endopeptidase activity, peptidase activity-acting on L-amino acid peptides, peptidase activity, catalytic activity, endopeptidase activity and hydrolase activity (Fig 3A, S8 Table). Then, a KEGG pathway enrichment analysis was performed and we found that the term metabolic pathways was the most significantly enriched (Fig 3B, S9 Table).

### Analysis of differentially expressed genes

**Fig 4:**
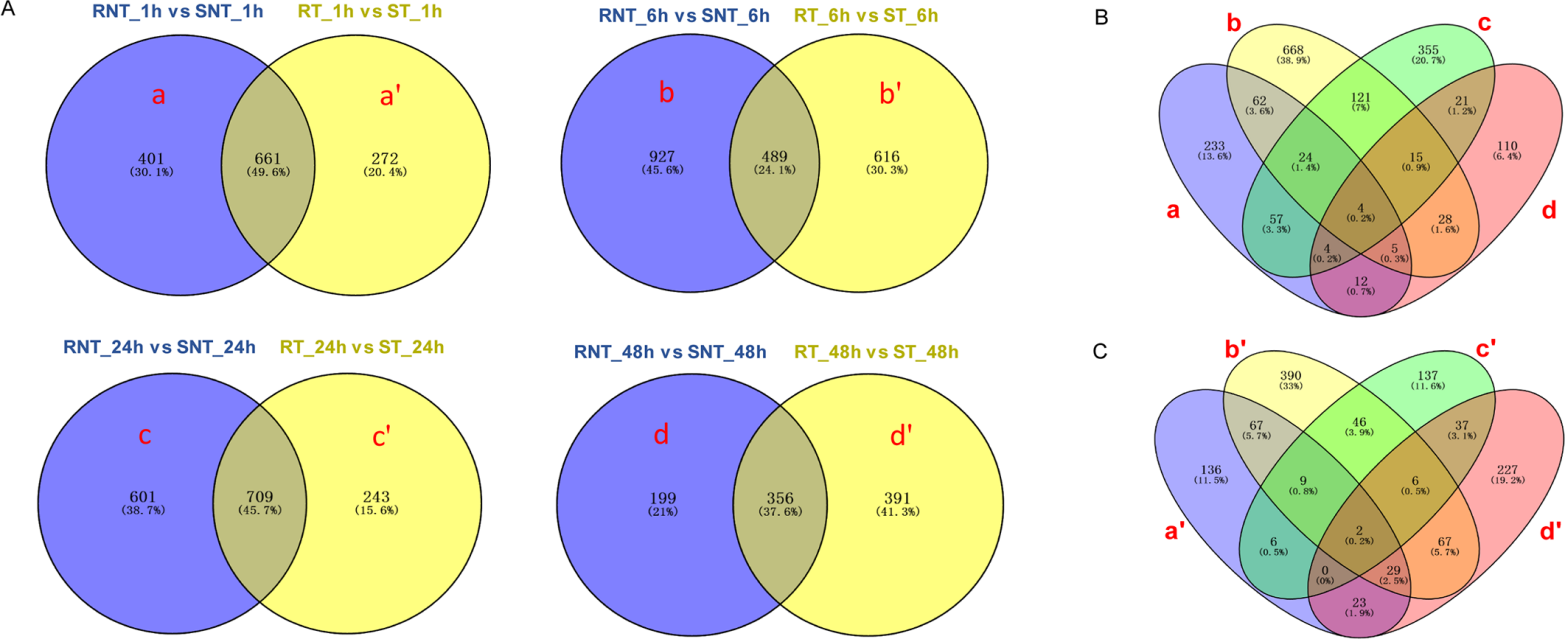
Venn diagrams of DEGs. (A) Venn diagram of DEGs in the two pairwise comparisons at different time points (1, 6, 24 and 48 h), respectively. a, b, c and d represent the unique DEGs in the RNT_1h vs SNT_1h, RNT_6h vs SNT_6h, RNT_24h vs SNT_24h and RNT_48h vs SNT_48h, respectively. a', b', c' and d' represent the unique DEGs in the RT_1h vs ST_1h, RT_6h vs ST_6h, RT_24h vs ST_24h and RT_48h vs ST_48h, respectively. (B) Venn diagram of the unique DEGs by comparing RNT_1h vs SNT_1h, RNT_6h vs SNT_6h, RNT_24h vs SNT_24h and RNT_48h vs SNT_48h. (C) Venn diagram of the unique DEGs by comparing RT_1h vs ST_1h, RT_6h vs ST_6h, RT_24h vs ST_24h and RT_48h vs ST_48h.

To investigate the major DEGs associated with the heat-humidity tolerance, the Venn diagram strategy was used to identify high temperature and humidity responsive DEGs at different times under 35 ± 0.5°C stress. In order to eliminate the interference from the genetic background of the two different silkworm strains, the Venn diagrams were constructed at different treatment times (Fig 4). First, we focused on DEGs that are responsive to high temperature and humidity. As shown in Fig 4A, there were 272 DEGs, 616 DEGs, 243 DEGs and 391 DEGs detected after excluding the same DEGs between the untreated groups at each time point. Then, another Venn diagram was constructed based on the DEGs from the above analysis (Fig 4C). As a result, we found that 6 genes, *BGIBMGA000387*, *BGIBMGA004579*, *BGIBMGA004675*, *BGIBMGA004910*, *BGIBMGA011699* and *BGIBMGA013893*, were differentially expressed in the two strains treated with high temperature and humidity for 6, 24 and 48 h (Table 2). Moreover, we found that the two genes, *BGIBMGA005701* and *BGIBMGA010163*, were differentially expressed in the two strains at all the treatment time points. On the other hand, to identify the differences between the control groups in both strains, we performed a Venn analysis based on DEGs obtained from the untreated groups at each time point (Fig 4B). Interestingly, 4 genes, *BGIBMGA003739*, *BGIBMGA005876*, *BGIBMGA011821* and *Novel01749*, were found to be differentially expressed between the two strains at all time points.

**Table 2:** Functional annotation of DEGs

Common Groups	Gene ID	Microarray probe	Putative function	Regulated
**RT_6h vs ST_6h RT_24h vs ST_24h RT_48h vs ST_48h**	BGIBMGA004675	sw21155	uncharacterized protein	up
	BGIBMGA004910	sw15361	transporter activity	up
	BGIBMGA000387	sw05903	uncharacterized protein	down
	BGIBMGA004579	sw18779	Calcium activated protein for secretion	down
	BGIBMGA011699	sw14638	uncharacterized protein	down
	BGIBMGA013893	sw17983	lipid transporter activity	down
**RT_1h vs ST_1h** **RT_6h vs ST_6h RT_24h vs ST_24h RT_48h vs ST_48h**	BGIBMGA005701	sw21526	response to wounding	up
	BGIBMGA010163	sw01449	GTPase activator activity	down
**RNT_1h vs SNT_1h** **RNT_6h vs SNT_6h RNT_24h vs SNT_24h RNT_48h vs SNT_48h**	BGIBMGA003739	sw10809	transmembrane transporter activity	down
	BGIBMGA005876	sw06369	Calcium ion binding	down
	BGIBMGA011821	sw06239	microtubule binding	down
	Novel01749	sw10286	serine-type peptidase activity	up

**Note:** DEGs were searched against the NBCI Non-redundant protein sequences (nr) database (https://blast.ncbi.nlm.nih.gov/Blast.cgi) and FlyBase (http://flybase.org/).

### Validation of DEGs by qRT-PCR

**Fig 5:**
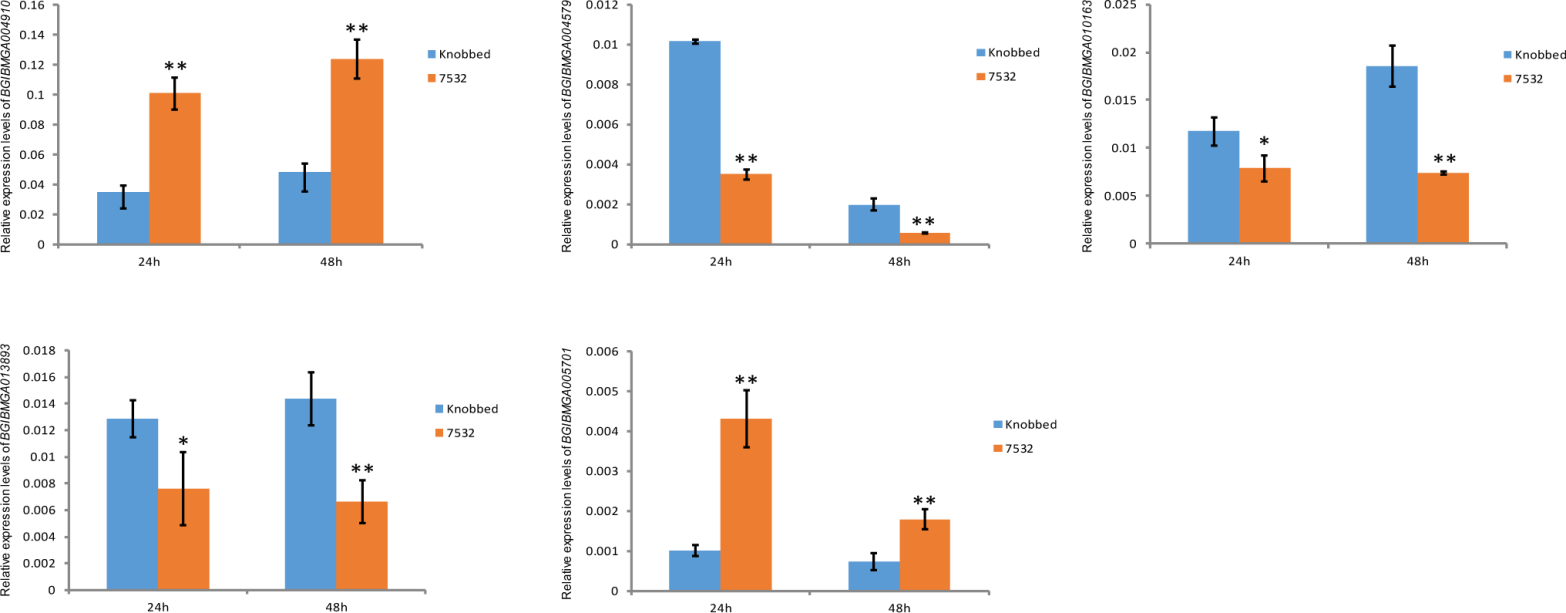
qRT-PCR validation of 5 identified DEGs with functional annotations. Bars indicate mean values ± SD (*n* = 3). *, *P* < 0.05; **, *P* < 0.01; Student's *t*-test.

To validate the transcriptome results, we selected 11 DEGs for confirmation by qRT-PCR analysis, including 5 identified DEGs with functional annotations (Table 2), 2 *Hsp* genes (*BGIBMGA004613* and *BGIBMGA004541*) and 4 randomly selected DEGs (*BGIBMGA009211*, *BGIBMGA007546*, *BGIBMGA005710* and *BGIBMGA000776*). The results of qRT-PCR showed up- or down-regulated gene expression profiles consistent with the RNA-seq data (Fig 5, S4 and S5 Figs, S7 and S10 Tables), indicating the reliability of the comparative analysis of our transcriptomes.

## Discussion

Tolerance of insects to high temperature and humidity is usually considered as an important trait for adaptation to the changing environment [8, 26–31]. Studies on the mechanisms of insect tolerance to high temperature and humidity are of great significance to control insect pests as well as to use economic insects efficiently. During the preservation and long-term investigation of silkworm characteristics, we found that the two silkworm strains, *7532* and *Knobbed*, were thermo-resistant and susceptible, respectively. These two strains with different resistance to high temperature and humidity present an excellent resource for identifying the key genes associated with the heat-humidity tolerance of silkworm. In this study, we performed multiple comparative transcriptome analyses of DEGs in these two strains during high temperature and humidity stress. Our data revealed important genes that respond to environmental stresses.

By investigating larval death in these two strains under high temperature and humidity stress, we found that the larvae of the susceptible strain, *Knobbed*, began to die from the second day with almost all dead on the fourth day (248/250). However, larvae of the resistant strain, *7532*, only had a few larval deaths (3/250) (Fig 1B, S2 Table). Interestingly, the results of DEGs analysis showed that the difference between RT_48h and RNT_48h was relatively large (Fig 2), indicating that the response of *7532* to high temperature and humidity was greater at 48 h than at other times. Based on these results, we presume that 48 h is a critical time point for silkworm to respond to the high temperature and humidity stress. Evidence from previous studies have indicated that high-temperature tolerance of insects can be effected at the cellular levels [4, 32]. In the present study, we identified 747 DEGs between RT_48h and ST_48h (S7 Table), and the GO and KEGG analysis showed that the metabolic process was significantly enriched (Fig 3), suggesting that the metabolic pathways play a significant role in the high temperature and humidity stress tolerance in silkworm [13]. Notably, our data showed three serine-related terms (serine-type peptidase activity, serine hydrolase activity, serine-type endopeptidase activity) to be significantly enriched (Fig 3A). Considering that serine is important for metabolism [33, 34], we presume that genes with serine-related enzyme activity may likely contribute to thermal tolerance of the silkworm.

Previous studies have reported that the heat shock proteins (Hsps), also known as stress proteins, may play a special role in the heat resistance of insects [4, 8, 35, 36]. Our data showed that several *Hsp* genes, such as *Hsp70* (*BGIBMGA004613*) and *sHsp20*.*4* (*BGIBMGA004541*), were up-regulated in the resistant strain, *7532*, after treatment for 24 and 48 h (S4 Fig), while the *sHsp19*.*5* (*BGIBMGA013545*) was down-regulated (S5 Fig, S7 and S10 Tables). These findings indicate that there may be differences in *Hsp* gene expression and function in response to high temperature and humidity. There is evidence of Hsps functions in dehydration tolerance in mosquitoes, especially the *Hsp 70* [37]. Moreover, considering that the sHsps are the first line of cellular defense in stressed cells [38, 39], *sHsp20*.*4* and *sHsp19*.*5* may be likely involved in protecting silkworms from exposure to high temperature and humidity stress.

Moreover, we identified eight common genes among the four pairs of comparisons with continuous high-temperature treatment (6, 24, 48 h or 1, 6, 24, 48 h). Three among these were up-regulated in resistant strains and the remaining five were down-regulated (Table 2), suggesting that these genes are particularly important for the heat-humidity stress response in the silkworm. Based on our homology searches, we found that the *Drosophila melanogaster* homologs of these eight genes (except for *BGIBMGA011699*) are all responsive to temperature or oxidative stress [40–42]. However, although several identified genes have been annotated with putative functions in biological metabolism, immune response and other processes, their roles in the heat-humidity stress response remain unclear and further studies are needed. In addition, given that thermal stress usually is associated with a general stop of transcription and a specific increase of transcriptional activity of the stress genes, we prefer genes that were up-regulated as candidates. Further, considering that the stress response is relatively fast, it is also important to understand the differences between the strains in the controls. We found that 4 genes were continually differentially expressed between the controls of the two strains (Table 2). Homology gene search showed that these genes are related to the maintenance of the internal milieu of insects. Hence, these genes were also considered to play a role in heat-humidity tolerance in silkworm.

## Supporting information

S1 Fig Hierarchical clustered graph of details of DEGs. SNT, *Knobbed* strain without high temperature and humidity treatment; ST, *Knobbed* strain with high temperature and humidity treatment; RNT, *7532* strain without high temperature and humidity treatment; RT, *7532* strain with high temperature and humidity treatment; the numbers after these sample names represent the time after treatment; h, hour.
(PDF)

S2 Fig A clustered graph based on the treated time between treated and non-treated of the same strain. SNT, *Knobbed* strain without high temperature and humidity treatment; ST, *Knobbed* strain with high temperature and humidity treatment; RNT, *7532* strain without high temperature and humidity treatment; RT, *7532* strain with high temperature and humidity and humidity treatment; the numbers after these sample names represent the time after treatment; h, hour.
(TIF)

S3 Fig Hierarchical clustered graph of details of DEGs corresponding to S2 Fig. SNT, *Knobbed* strain without high temperature and humidity treatment; ST, *Knobbed* strain with high temperature and humidity treatment; RNT, *7532* strain without high temperature and humidity treatment; RT, *7532* strain with high temperature and humidity and humidity treatment; the numbers after these sample names represent the time after treatment; h, hour.
(PDF)

S4 Fig qRT-PCR validation of DEGs. Bars indicate mean values ± SD (*n* = 3). *, *P* < 0.05; **, *P* < 0.01; Student's *t*-test.
(TIF)

S5 Fig FPKM fold changes of sequencing results. (TIF)

S1 Table Information of primers used in this work. (DOCX)

S2 Table Investigation and statistics of the death of 7532 and Knobbed strains. (DOCX)

S3 Table Summary of RNA-seq data mapped to the silkworm reference genome. (XLSX)

S4 Table Novel genes identified according to the gene model annotation file. (XLSX)

S5 Table Distribution of transcript levels in the 16 samples. (XLSX)

S6 Table Expression levels of all the DEGs among the 16 samples by comparing any two samples (XLSX)

S7 Table DEGs identified between RT_48h and ST_48h (XLSX)

S8 Table Gene Ontology (GO) classification of the DEGs obtained from the comparison RT_48h vs ST_48h (XLSX)

S9 Table KEGG classification of the DEGs obtained from the comparison RT_48h vs ST_48h (XLSX)

S10 Table DEGs identified between RT_24h and ST_24h (XLSX)
