# Supplementary figures and images for: Comparative transcriptome profiling of a thermal resistant vs. sensitive silkworm strain in response to high temperature under stressful humidity condition
Source: PLoS One. 2017 May 18;12(5):e0177641. doi: 10.1371/journal.pone.0177641 (PMC5436693; doi:10.1371/journal.pone.0177641)

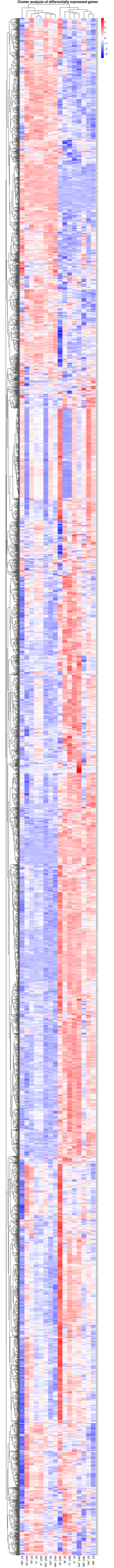

Supplement: S1 Fig — SNT, Knobbed strain without high temperature and humidity treatment; ST, Knobbed strain with high temperature and humidity treatment; RNT, 7532 strain without high temperature and humidity treatment; RT, 7532 strain with high temperature and humidity treatment; the numbers after these sample names represent the time after treatment; h, hour. (PDF) [file pone.0177641.s001.pdf]

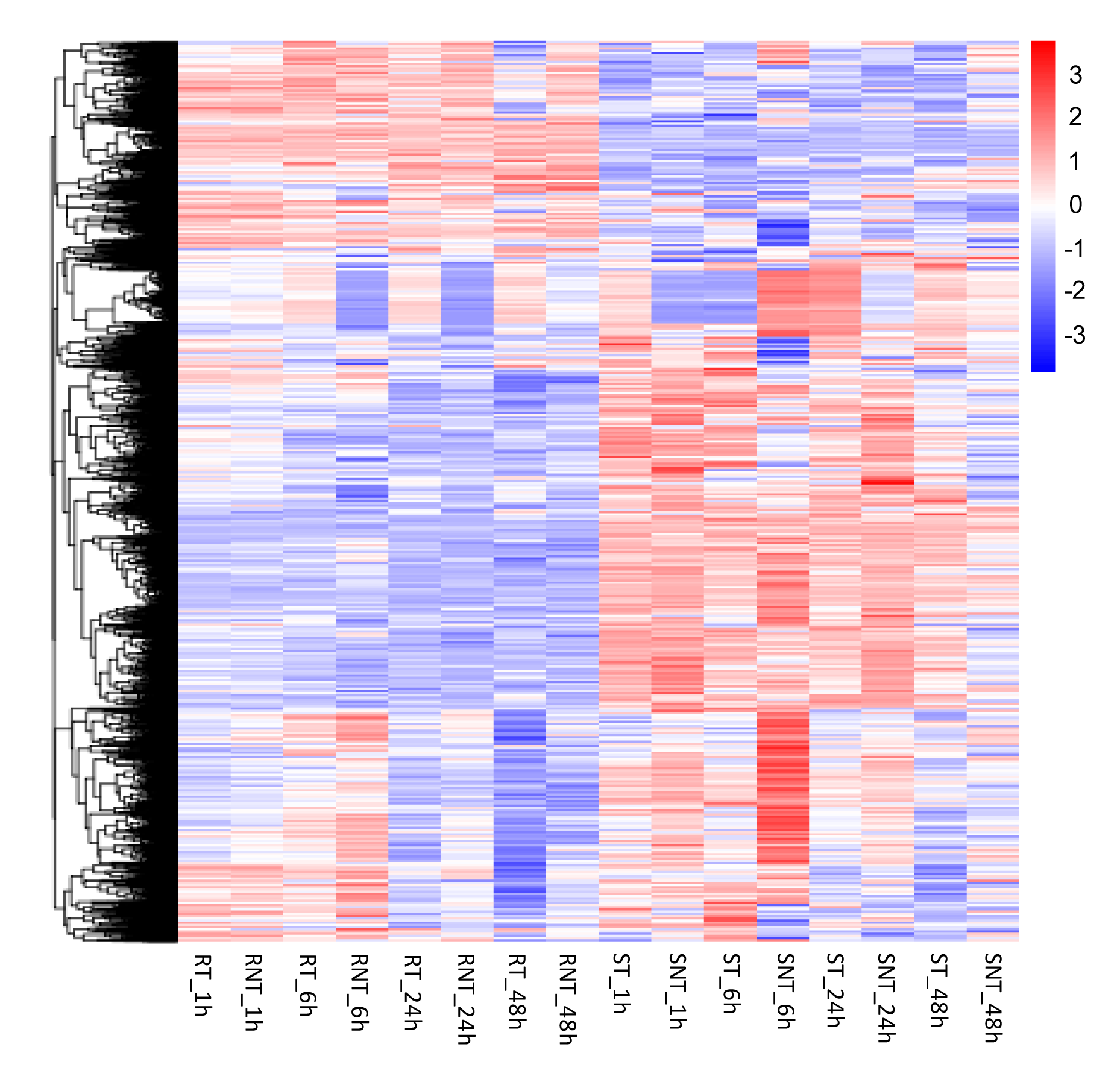

Supplement: S2 Fig — SNT, Knobbed strain without high temperature and humidity treatment; ST, Knobbed strain with high temperature and humidity treatment; RNT, 7532 strain without high temperature and humidity treatment; RT, 7532 strain with high temperature and humidity and humidity treatment; the numbers after these sample names represent the time after treatment; h, hour. (TIF) [file pone.0177641.s002.tif]

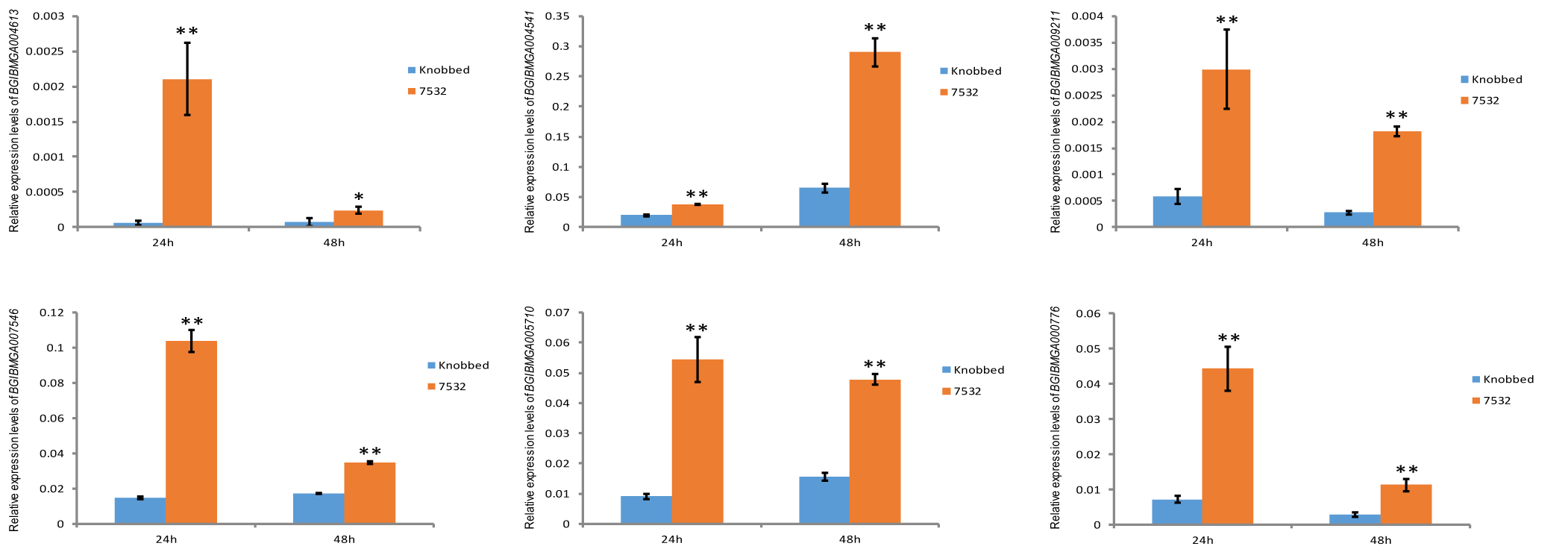

Supplement: S4 Fig — Bars indicate mean values ± SD (n = 3). *, P < 0.05; **, P < 0.01; Student's t-test. (TIF) [file pone.0177641.s004.tif]

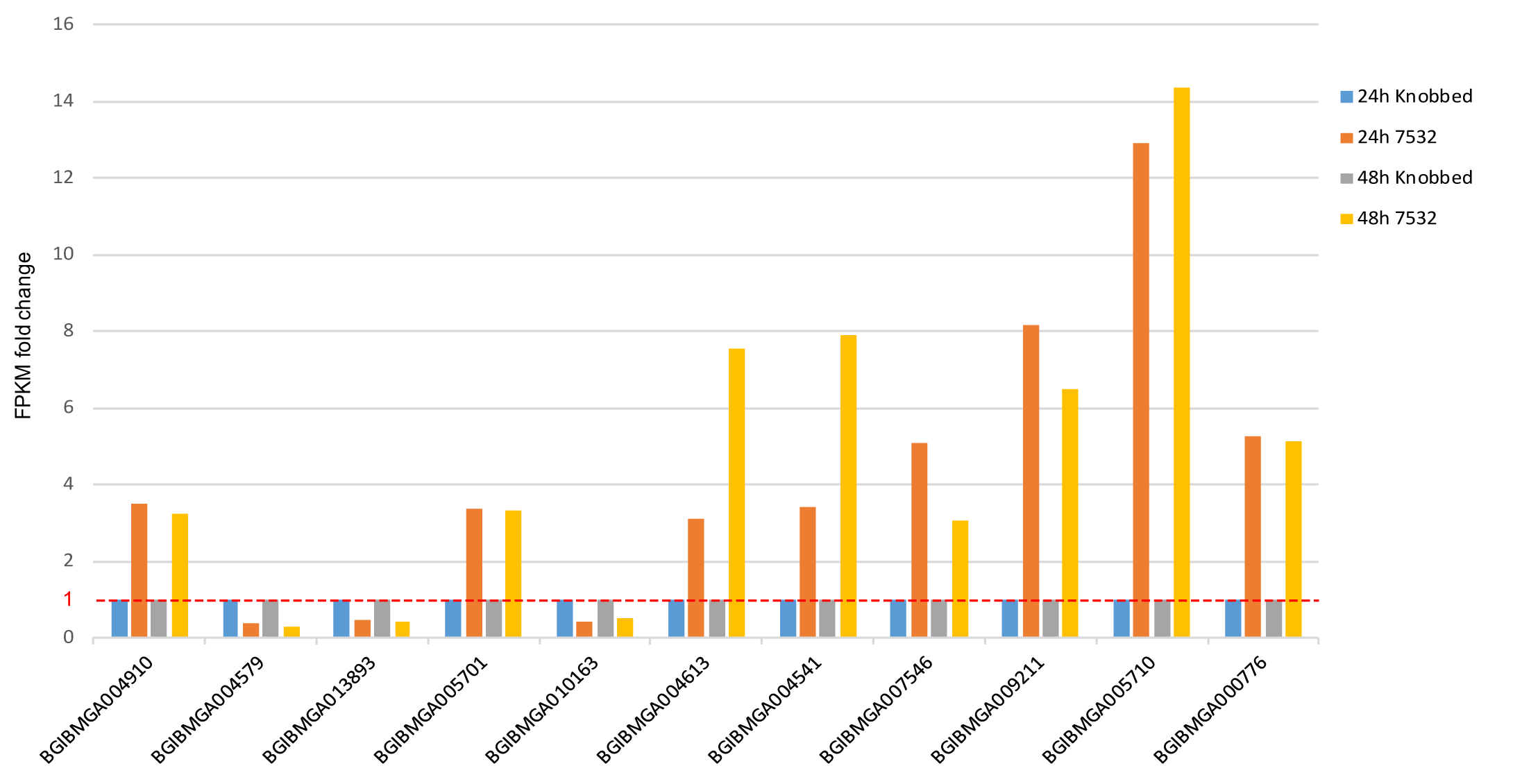

Supplement: S5 Fig — (TIF) [file pone.0177641.s005.tif]
