# Supplementary material for: Comparative transcriptome profiling of a thermal resistant vs. sensitive silkworm strain in response to high temperature under stressful humidity condition
Source: PLoS One. 2017 May 18;12(5):e0177641. doi: 10.1371/journal.pone.0177641 (PMC5436693; doi:10.1371/journal.pone.0177641)
Supplement: S1 Table — (DOCX) [file pone.0177641.s006.docx]

**S1 Table. Information of primers used in this work**

| **Primer name** | **Sense sequence (5′−3′)** | **Antisense sequence (5′−3′)** | **Amplicon size** | **Primer efficiency** |
| --- | --- | --- | --- | --- |
| *BGIBMGA004910* | GGTCAGCACGGAGACTTGGA | ACATTGGAGCCGCTTTATCTTT | 229 bp | 99.27% |
| *BGIBMGA004579* | ATTACGGGACAAAGTCACAAACC | TAAGTAGTCCTCCATTCCGTGCT | 108 bp | 90.21% |
| *BGIBMGA013893* | CCGACGCACCATACTTCCTAA | CACAAACTCGCCTCCAACG | 304 bp | 90.75% |
| *BGIBMGA005701* | CGTCACAGCGAACCAAGATG | CCACCTGGATGGTCTCGTAGTT | 407 bp | 104.27% |
| *BGIBMGA010163* | AAGAGTGACTTTAGCGATGGTGC | TCGGTTGGGAACTTGAATGAC | 197 bp | 95.42% |
| *BGIBMGA004613* | GAAGCCTATCTGGGAAAGCG | TCCTCGCTTGTTGGTATCCTC | 346 bp | 90.48% |
| *BGIBMGA004541* | CGTCTGTTGGACCAGCATTTT | CGGACGAACTGCCTTGAAAT | 296 bp | 104.21% |
| *BGIBMGA007546* | ACGAGAGGTTACAAGGACGACAC | AATGTTGGCGGTGGTAAAGGT | 113 bp | 90.47% |
| *BGIBMGA009211* | AGCGGCAGAATACCTACTCCAG | GGCTCTCCATTACCGTGACCT | 239 bp | 103.16% |
| *BGIBMGA005710* | TGGATGCTGCCGCTACAAG | GACCACCTCCAACGACTACGA | 150 bp | 95.99% |
| *BGIBMGA000776* | ATGCCAATGTCTTTGTGCCC | TCTCTTTGAACCCAGCGTAGC | 283 bp | 103.36% |
| *sw22934* | TTCGTACTGGCTCTTCTCGT | CAAAGTTGATAGCAATTCCCT | 174 bp | 93.96% |
