# Supplementary material for: Comparative transcriptome profiling of a thermal resistant vs. sensitive silkworm strain in response to high temperature under stressful humidity condition
Source: PLoS One. 2017 May 18;12(5):e0177641. doi: 10.1371/journal.pone.0177641 (PMC5436693; doi:10.1371/journal.pone.0177641)
Supplement: S2 Table — (DOCX) [file pone.0177641.s007.docx]

**S2 Table. Investigation and statistics of the death of *7532* and *Knobbed* strains.**

| Strains | | Death Statistics | | | | | | | Total |
| --- | --- | --- | --- | --- | --- | --- | --- | --- | --- |
| Group | Replicates | Day 1 | Day 2 | Day 3 | Day 4 | Day 5 | Day 6 | Day 7 |  |
| *Knobbed*（Non-Treated） | 1 | 0 | 1 | 3 | 3 | 5 | 4 | 7 | 23 |
|  | 2 | 0 | 0 | 2 | 4 | 4 | 3 | 5 | 18 |
|  | 3 | 0 | 0 | 4 | 5 | 3 | 5 | 5 | 22 |
|  | 4 | 0 | 0 | 2 | 3 | 3 | 5 | 3 | 16 |
|  | 5 | 0 | 0 | 3 | 4 | 4 | 5 | 5 | 21 |
|  | Total | 0 | 1 | 14 | 19 | 19 | 22 | 25 | 100 |
| *Knobbed*（Treated） | 1 | 0 | 25 | 14 | 11 | / | / | / | 50 |
|  | 2 | 0 | 23 | 18 | 9 | / | / | / | 50 |
|  | 3 | 0 | 19 | 21 | 10 | / | / | / | 50 |
|  | 4 | 0 | 17 | 22 | 11 | / | / | / | 50 |
|  | 5 | 0 | 21 | 18 | 9 | 2 | / | / | 50 |
|  | Total | 0 | 105 | 93 | 50 | 2 | 0 | 0 | 250 |
| *7532*（Non-Treated） | 1 | 0 | 0 | 0 | 0 | 0 | 0 | M | 0 |
|  | 2 | 0 | 1 | 0 | 0 | 0 | 0 | M | 1 |
|  | 3 | 0 | 0 | 0 | 0 | 0 | 0 | M | 0 |
|  | 4 | 0 | 0 | 0 | 1 | 0 | 0 | M | 1 |
|  | 5 | 0 | 0 | 0 | 0 | 0 | 2 | M | 2 |
|  | Total | 0 | 1 | 0 | 1 | 0 | 2 | 0 | 4 |
| *7532*（Treated） | 1 | 0 | 0 | 0 | 0 | 1 | M | M | 1 |
|  | 2 | 0 | 0 | 0 | 0 | 0 | M | M | 0 |
|  | 3 | 0 | 0 | 0 | 2 | 0 | M | M | 2 |
|  | 4 | 0 | 0 | 0 | 0 | 0 | M | M | 0 |
|  | 5 | 0 | 0 | 0 | 0 | 0 | M | M | 0 |
|  | Total | 0 | 0 | 0 | 2 | 1 | 0 | 0 | 3 |

Note: M represents mounting.
