# Supplementary material for: Comparative transcriptome profiling of a thermal resistant vs. sensitive silkworm strain in response to high temperature under stressful humidity condition
Source: PLoS One. 2017 May 18;12(5):e0177641. doi: 10.1371/journal.pone.0177641 (PMC5436693; doi:10.1371/journal.pone.0177641)
Supplement: S3 Fig — SNT, Knobbed strain without high temperature and humidity treatment; ST, Knobbed strain with high temperature and humidity treatment; RNT, 7532 strain without high temperature and humidity treatment; RT, 7532 strain with high temperature and humidity and humidity treatment; the numbers after these sample names represent the time after treatment; h, hour. (PDF) [file pone.0177641.s003.pdf]

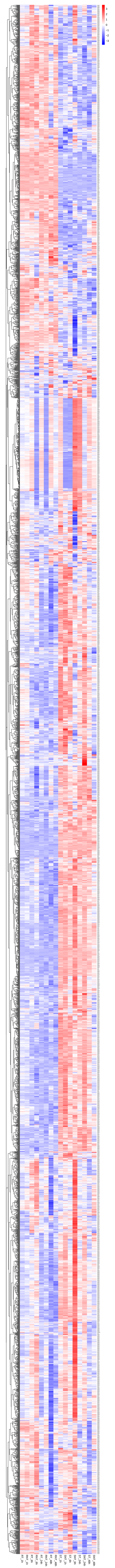

3
2
1
0
-1
-2
-3
RT_1h
RNT_1h
RT_6h
RNT_6h
RT_24h
RNT_24h
RT_48h
RNT_48h
ST_1h
SNT_1h
ST_6h
SNT_6h
ST_24h
SNT_24h
ST_48h
SNT_48h
